# The Pepper Late Embryogenesis Abundant Protein, CaDIL1, Positively Regulates Drought Tolerance and ABA Signaling

**DOI:** 10.3389/fpls.2018.01301

**Published:** 2018-09-04

**Authors:** Junsub Lim, Chae Woo Lim, Sung Chul Lee

**Affiliations:** Department of Life Science (BK21 Program), Chung-Ang University, Seoul, South Korea

**Keywords:** abscisic acid, drought, late embryogenesis abundant, stomata, transpiration, virus-induced gene silencing

## Abstract

Plants as sessile organisms constantly respond to environmental stress during their growth and development. The regulation of transpiration via stomata plays crucial roles in plant adaptation to drought stress. Many enzyme-encoding genes are involved in regulation of transpiration via modulating stomatal opening and closure. Here, we demonstrate that *Capsicum annuum* Drought Induced Late embryogenesis abundant protein 1 (*CaDIL1*) gene is a critical regulator of transpirational water loss in pepper. The expression of *CaDIL1* in pepper leaves was upregulated after exposure to abscisic acid (ABA) and drought. Phenotype analysis showed that *CaDIL1*-silenced pepper and *CaDIL1-o*verexpressing (OX) Arabidopsis transgenic plants exhibited reduced and enhanced drought tolerance, respectively, accompanied by an altered water loss. Furthermore, ABA sensitivity was significantly lower in *CaDIL1*-silenced pepper, but higher in *CaDIL1*-OX plants, than that in control plants, which resulted in opposite responses to drought stress in these two plant types. Collectively, our data suggest that *CaDIL1* positively regulates the ABA signaling and drought stress tolerance.

## Introduction

Drought is one of environmental stresses that inhibit plant growth and significantly affects agricultural crop productivity (Zhu, 2002; Sengupta and Majumder, 2010). Plants are sessile organisms and frequently encountered to drought stress, which causes osmotic stress and leads to serious damage to plant tissues. To adapt to drought stress, plants display physiological and molecular changes, including stomatal closure and reprogramming of gene expression (Krasensky and Jonak, 2012, Lee and Luan, 2012). The adaptive mechanisms underlying functional modifications has not been fully understood, because the mechanisms complex and diverse at the cellular level as well as at the organism level (Ding et al., 2015; Lim et al., 2015a; Zou et al., 2015).

Abscisic acid (ABA) is a major plant hormone that plays a key role in cellular adaptive response to drought stress. The biosynthesis and accumulation of ABA is enhanced in drought conditions, and these initiate plant adaptive responses (Hubbard et al., 2010). A large number of genes are regulated by ABA and associated with ABA signal transduction pathway; for example, ABA induces more than 10% of Arabidopsis genes (Goda et al., 2008; Mizuno and Yamashino, 2008). Until recent studies, ABA affects the stomatal aperture via modulation of cation and anion channels from guard cells leading to inhibition of transpirational water loss, which is indispensable to plant survival in drought stress (Geiger et al., 2009; Lee et al., 2009). Therefore, ABA-deficient mutants showed hypersensitive phenotype to drought stress (Vlad et al., 2009; Umezawa et al., 2013; Zou et al., 2015; Baek et al., 2017). In contrast, mutants that exhibit ABA hypersensitive phenotype display drought tolerance (Lim et al., 2017). Moreover, a large number of genes involved in the adaptive response to drought stress are modulated by ABA (Shinozaki and Yamaguchi-Shinozaki, 2007; Lim et al., 2015b).

The late embryogenesis abundant (LEA) proteins are present in bacteria, yeast, fungi, and many species of vascular plants (Hong-Bo et al., 2005). LEA proteins function in a large spectrum of cellular processes, from growth to stress response (Xiao et al., 2007; Campos et al., 2013; Pathak and Ikeno, 2017). In vascular plants, LEA proteins are highly expressed in embryos during the late embryogenesis stage and in vegetative organs under drought stress conditions, indicating that these proteins play an adaptive role in water limitation (Dure and Chlan, 1981; Baker et al., 1988; Lu et al., 2018). Previous studies showed that some LEA proteins are involved in ABA signaling and protect cells against stresses, especially osmotic stress (Hu et al., 2008; Candat et al., 2014; Lim et al., 2015c). For instance, overexpression of rice OsLEA5 (Huang et al., 2018) confers tolerance to drought and high salinity, and LEA3 (Xiao et al., 2007) and RAB16D (Tang et al., 2016) are involved in adaptive response to water deficit conditions. These proteins are composed of hydrophilic amino acids and constitute multigene families (Dure et al., 1989), which are classified into seven groups based on their amino acid sequence similarity and corresponding mRNA homology (Hong-Bo et al., 2005; Battaglia et al., 2008). Group 3 comprises typical LEA proteins, as they exhibit hydrophilic characteristics and are considerably more diverse than other groups of LEA proteins (Battaglia et al., 2008). Although the precise biological role of group 3 LEA proteins have not been fully understood, previous studies elucidated that these genes are induced by drought stress in several non-plant organisms as well as in plants (Hsing et al., 1995; Romo et al., 2001; Gal et al., 2004; Hand et al., 2007; Battaglia et al., 2008).

In our previous study, we isolated and characterized a group 5 LEA protein gene, *CaLEA1*, which is involved in the adaptive response to drought and high salinity (Lim et al., 2015c). Here, we identified and characterized a novel pepper group 3 LEA protein gene, *CaDIL1* (*Capsicum annuum* Drought Induced Late embryogenesis abundant protein 1). We isolated this gene from a pepper using RNA-seq analysis and examined the *in vivo* function of CaDIL1 in *CaDIL1*-silenced pepper and *CaDIL1*-overexpressing (OX) transgenic *Arabidopsis* plants.

## Materials and Methods

### Virus-Induced Gene Silencing and Overexpression of *CaDIL1*

For the loss-of-function analysis of CaDIL1, we used the tobacco rattle virus (TRV)-based VIGS system to generate *CaDIL1* gene knockdown in pepper according to the protocol described by Lim et al. (2017). We used a 247 bp (116–362 nucleotide sequences) and the full length of *CaDIL1* cDNA to generate *CaDIL1*-silenced pepper and *CaDIL1*-overexpressing (OX) *Arabidopsis thaliana*, respectively, as described previously (Park et al., 2015).

### Subcellular Localization of GFP-Tagged CaDIL1 Protein

*Agrobacterium tumefaciens* strain GV3101 carrying the 35S promoter-driven *CaDIL1-GFP* gene construct was combined with strain p19 (1:1 ratio; OD_600_ = 0.5) and co-infiltrated into the abaxial side of leaves of 4-week-old *Nicotiana benthamiana* plants. At 2 days after infiltration, the GFP signal was observed under a confocal microscope (510 UV/Vis Meta; Zeiss, Oberkochen, Germany) equipped with LSM Image Browser software.

### Treatments of ABA and Drought Stress

To estimate ABA sensitivity, we measured the germination rate, root elongation, and seedling establishment. A total of 200 seeds each of both plants were stratified at 4°C for 2 days and plated on 0.5 × MS agar medium with ABA. For drought stress treatment in pepper and Arabidopsis, drought stress was imposed on was imposed on four-leaf stage and 3-week-old seedlings, respectively, by withholding watering and re-watering for recovery. All the experiments were conducted at three times.

### Measurement of Transpirational Water Loss

Transpirational water loss was measured as described previously (Lim et al., 2017). Briefly, fully expanded first and second leaves were detached from four-leaf-stage pepper plants, and four rosette leaves were harvested from 3-week-old Arabidopsis plants (*n* = 15 plants per line). The detached leaves were placed in Petri dishes and their fresh weights were measured at hourly intervals. All the experiments were conducted at three times.

### Stomatal Aperture Bioassay and Thermal Imaging

To measure stomatal aperture, fresh leaves of pepper and *Arabidopsis* were harvested. The epidermal peels were incubated by floating in stomatal opening buffer (50 mM KCl and 10 mM MES-KOH, pH 6.15, 10 mM CaCl_2_) with or without ABA at 24°C for 2.5 h. Photographs were taken under a Nikon Eclipse 80i microscope. To measure the leaf temperature, thermal images were taken by an infrared camera (T420; FLIR systems) of 4-week-old pepper and 3-week-old Arabidopsis treated with 0 or 100 μM ABA. Leaf temperatures were measured by using FLIR Tools+ ver 5.2 software.

### Real-Time Transcription-Polymerase Chain Reaction

cDNA was synthesized with total RNA extracted from pepper and Arabidopsis by a Transcript First Strand cDNA Synthesis kit (Roche). For real time-PCR assays, the specific primers (**Supplementary Table S1**) and CFX96 Touch^TM^ Real-Time PCR detection system (Bio-Rad) were used. The PCR was programmed as follows: 95°C for 5 min; 45 cycles each at 95°C for 20 s and 60°C for 20 s; and 72°C for 20 s. To determine relative expression level, we used the ΔΔCt method (Livak and Schmittgen, 2001). *Arabidopsis* *AtACT8* gene was used for normalization.

## Results

### Isolation and Sequence Analysis of the Pepper *CaDIL1* Gene

**FIGURE 1:**
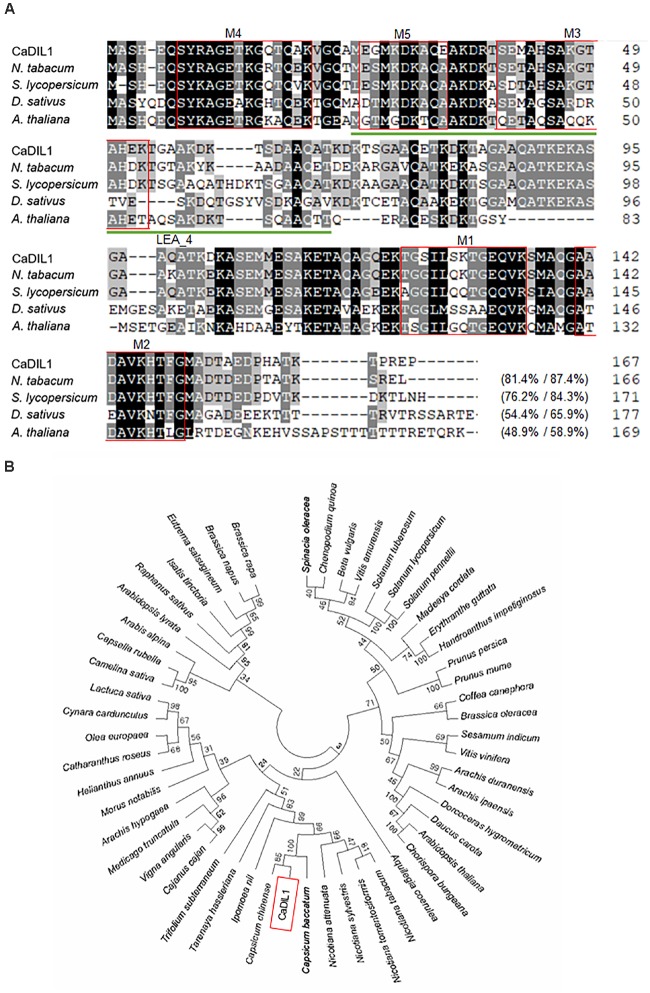
Homology of the pepper CaDIL1 (*Capsicum* *annuum* Drought Induced Late embryogenesis abundant protein 1) protein with LEA3 proteins. **(A)** Comparisons of the deduced CaDIL1 amino acid sequence with those of *Nicotiana tabacum* (accession no. XP_016459037.1), *Solanum lycopersicum* (accession no. NP_001238798.1), *Daucus* *sativus* (accession no. XP_017256990.1), and *Arabidopsis thaliana* (accession no. NP_175678.1) proteins. Identical amino acid residues are highlighted in black. Multiple alignment of the CaDIL1 protein sequence and its homologous LEA3 proteins was performed using ClustalW2. Red boxes and green underline indicate repetitive motifs and LEA_4 Pfam domain of group 3 LEA proteins. **(B)** Phylogenetic tree analysis of CaDIL1 protein. BLAST search was performed by using deduced amino acid sequences of *CaDIL1*, and sequences with highest similarity were gathered from each plant species. The phylogenetic tree was generated using MEGA software (version 7.0).

To identify a drought-induced gene, we used RNA-seq analysis and isolated the pepper *CaDIL1* gene from drought-treated pepper leaves (Lim and Lee, 2016). The *CaDIL1* cDNA consists of an open reading frame of a 504 bp nucleic acid residue that codifies for a 167-amino acid residue with an isoelectric point of 7.89 and a molecular weight of 17,300 Da. As shown in **Figure 1A**, CaDIL1 contains LEA_4 Pfam domain (PF02987) and 5 repetitive motifs, which are characterized in Group 3 LEA proteins (Battaglia et al., 2008). Protein BLAST search and multiple sequence alignment analysis, conducted to identify sequences with highest similarities, revealed relatively high amino acid sequence identity (48.9–81.4%) between CaDIL1 and other LEA proteins (**Figure 1A**). As predicted, phylogenetic analyses revealed that CaDIL1 clustered with Group 3 LEA proteins from various flowering plants, especially a family of *Solanaceae* (**Figure 1B**).

### Induction of *CaDIL1* in Response to Drought and ABA Treatments and Subcellular Localization of *CaDIL1*

**FIGURE 2:**
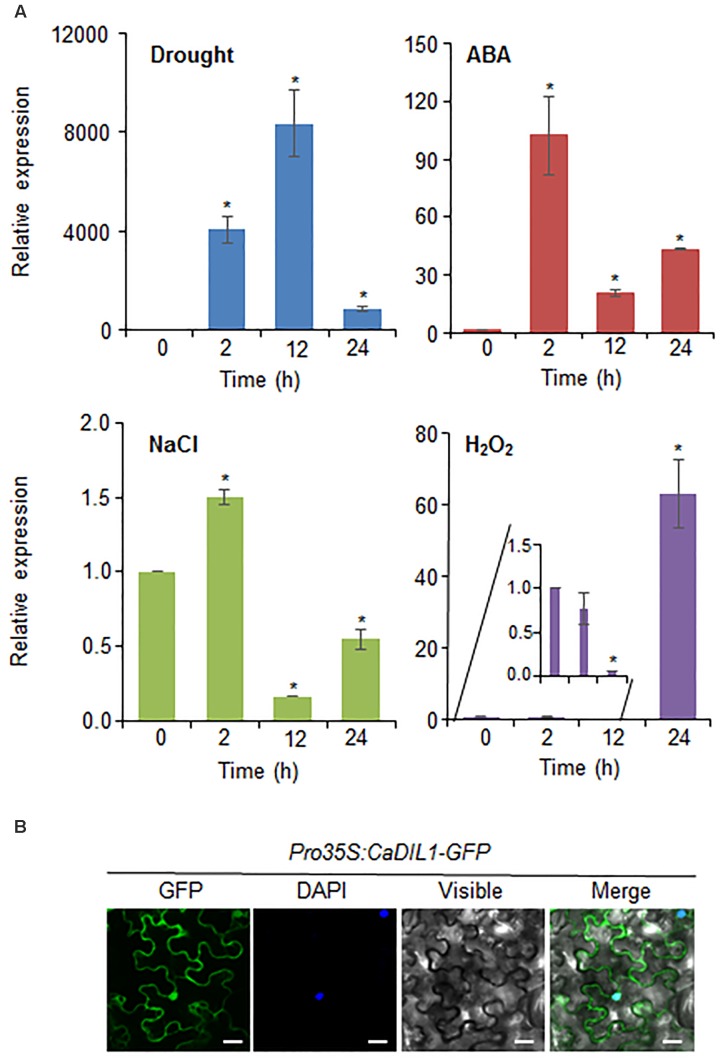
∣ Expression of the *CaDIL1* gene and localization of the CaDIL1 protein. **(A)** Induction of *CaDIL1* in pepper leaves at various time points after treatment with drought, 50 μM abscisic acid (ABA), 200 mM NaCl and 100 μM H_2_O_2_. The pepper *Actin1* genes were used as internal control. Data represent the mean ± standard error of five independent experiments. Asterisks indicate significant differences between 0 h and other times (Student’s *t*-test; *P* < 0.05). **(B)** Subcellular localization of the CaDIL1 protein using transient expression of the green fluorescent protein (GFP) fusion protein in *Nicotiana benthamiana* cells. The 35S:*CaDIL1-GFP* construct was expressed using agroinfiltration of *N. benthamiana* leaves and observed under a confocal laser-scanning microscope. 4′,6-Diamidino-2-phenylindole (DAPI) staining was used as a marker for the nucleus. White bar = 20 μm.

Since *CaDIL1* was isolated from drought-treated pepper leaves, we examined the induction levels of *CaDIL1* after drought, ABA, NaCl, and H_2_O_2_ treatments (**Figure 2A**). The induction of *CaDIL1* transcripts was induced at 2 h and increased level at 12 h after drought stress. It is well known the function of ABA involved in drought stress response, and ABA and drought stress share common signaling pathways (Jakab et al., 2005). After ABA treatment, *CaDIL1* transcription started at 2 h and decreased at 12 h after ABA treatment. Moreover, the *CaDIL1* transcripts accumulated more strongly by treatment of high salinity. H_2_O_2_ plays a role as signal molecule in ABA-induced stomatal closure, we investigated the expression level of *CaDIL1* after H_2_O_2_ treatments. As shown in **Figure 2A**, the expression level of *CaDIL1* is induced by H_2_O_2_. These results suggest that *CaDIL1* functions in plant responses to drought, high salinity, and ROS.

To examine the subcellular localization of CaDIL1 in the cells, we fused the *CaDIL1* cDNA with green fluorescent protein (GFP) (*35S:CaDIL1-GFP*). The CaDIL1 protein was expressed in epidermal cells of *Nicotiana benthamiana* and GFP signals were localized in the cytoplasm and nucleus (**Figure 2B**). The DAPI signal of blue fluorescent was expressed in nucleus. These results suggest that CaDIL1 plays a role in the cytoplasm and nucleus.

### Reduced Tolerance of *CaDIL1*-Silenced Pepper Plants to Drought Stress

**FIGURE 3:**
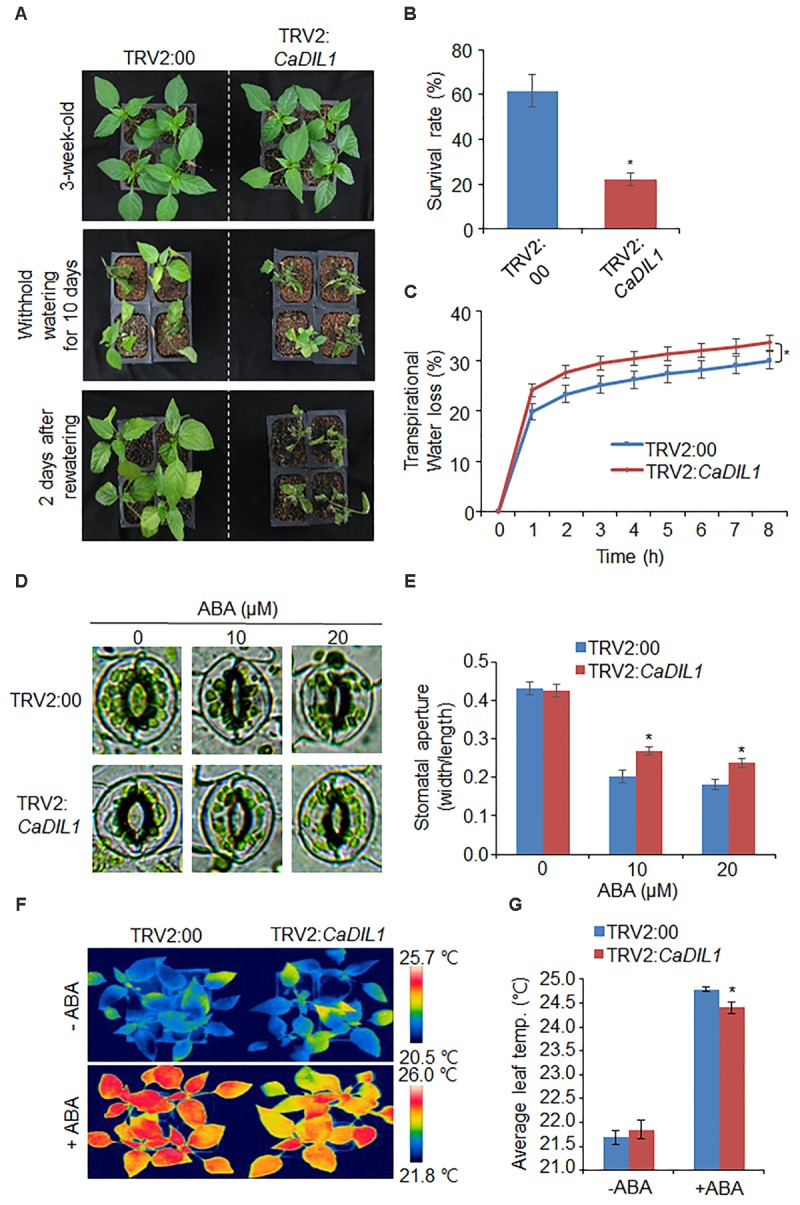
Reduced tolerance of *CaDIL1*-silenced pepper plants to drought stress. **(A)** Drought susceptibility of *CaDIL1*-silenced pepper plants. Empty vector control and *CaDIL1*-silenced pepper plants were grown in pots for 3 weeks under normal growth conditions. The plants were then subjected to drought stress by withholding watering for 10 days, followed by rewatering for 2 days. Representative images were taken before (left) and after (middle) dehydration, and 2 days after rewatering (right). **(B)** The survival rate was measured by counting the number of plants with green and rehydrated leaves 2 days after rewatering. Data represent the mean ± standard error of three independent experiments, each evaluating 30 plants. **(C)** Transpirational water loss from the leaves of empty vector control and *CaDIL1*-silenced pepper plants at various times after detachment of leaves. Data represent the mean ± standard deviation of three independent experiments, each evaluating 50 leaves. **(D,E)** Stomatal apertures in empty vector control and *CaDIL1*-silenced pepper plants after abscisic acid (ABA) treatment. Representative images were taken under a microscope **(D)** and the stomatal apertures were measured **(E)**. Leaf peels were harvested from 2-week-old pepper plants and incubated in stomatal opening solution containing 10 and 20 μM ABA; the stomatal apertures were then measured under a microscope. Representative images were taken before (left) and 2.5 h after (right) ABA treatment. Data represent the mean ± standard error of three independent experiments, each evaluating 20 plants. **(F,G)** Decreased leaf temperatures of *CaDIL1*-silenced pepper plants after ABA treatment. Representative images were taken **(F)** and the leaf temperatures were measured **(G)**. Data represent the mean ± standard error of three independent experiments, each evaluating 10 plants. Asterisks indicate significant differences between the control and the *CaDIL1*-silenced pepper plants (Student’s *t*-test; *P* < 0.05).

As *CaDIL1* was isolated from drought-treated pepper leaves, we postulated that *CaDIL1* is associated with drought response. To test this, we examined phenotype assays using VIGS analysis in pepper and generated overexpressing *Arabidopsis thaliana*. First, we checked the gene silencing using RT-PCR analysis of control (TRV2:00) and *CaDIL1*-silenced pepper (TRV2:*CaDIL1*) plants. The *CaDIL1* expression level in the *CaDIL1*-silenced pepper leaves was lower than in control plants (**Figure 3A**) and these plants were used in subsequent phenotypic assays. We treated the *CaDIL1*-silenced pepper plants and control plants with drought stress and compared their phenotypes (**Figure 3B**). Under normal growth conditions, we did not observe any phenotypic differences in both plants (**Figure 3B**, upper panel). However, when we treated the both plants to drought stress by withholding watering for 10 days and then rewatered them for 2 days, *CaDIL1*-silenced pepper plants displayed a more wilted phenotype than control plants (**Figure 3B**, middle and lower panel). The survival rates of *CaDIL1*-silenced pepper plants and control plants were 22.2 and 61.7%, respectively. On the basis of the drought-sensitive phenotype exhibited by *CaDIL1*-silenced pepper plants, we postulated that water retention capacity was reduced in *CaDIL1*-silenced pepper plants. To test this hypothesis, we measured the transpirational water loss in the leaves of control plants and *CaDIL1*-silenced pepper plants (**Figure 3C**). After detachment, the fresh weight was lower in the leaves of *CaDIL1*-silenced pepper plants than in those of control plants. To evaluate whether the decreased water retention displayed by *CaDIL1*-silenced pepper leaves was derived from a reduced sensitivity to ABA, we monitored stomatal apertures and leaf temperatures with or without ABA treatment (**Figures 3D–G**). In the absence of ABA, stomatal apertures were not significant differences in both plants (**Figures 3D,E**). However, the stomatal pore sizes were larger in the *CaDIL1*-silenced pepper than in control plants after ABA treatment. Consistent with stomatal aperture, the leaf temperatures of *CaDIL1*-silenced pepper were lower than those of control plants in the presence of ABA (**Figures 3F,G**) due to evaporative cooling caused by open stomata. Collectively, these data indicate that the high rate of transpiration of *CaDIL1*-silenced pepper plants, which leads to enhanced drought sensitivity, is mainly owing to reduced ABA sensitivity.

### Enhanced Sensitivity of *CaDIL1*-OX Plants to ABA

**FIGURE 4:**
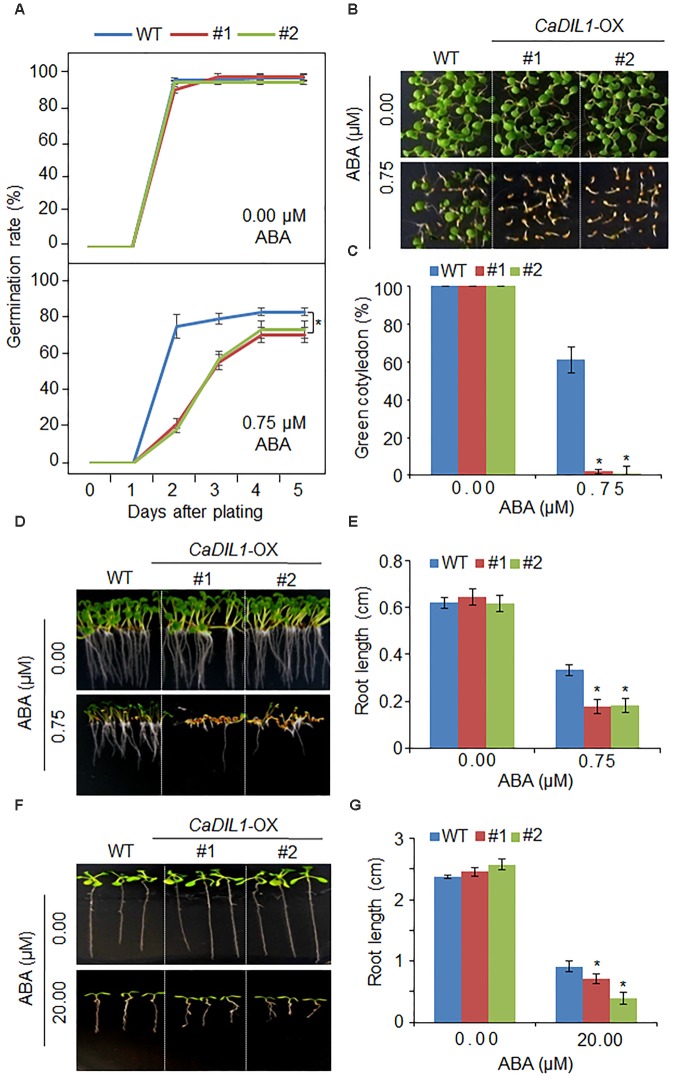
Enhanced sensitivity of *CaDIL1*-OX transgenic *Arabidopsis* lines to abscisic acid (ABA). **(A)** Germination rates of *CaDIL1*-OX mutants and wild-type (WT) plants on 0.5 × Murashige and Skoog (MS) medium supplemented with various concentrations of ABA. Data represent the mean ± standard error of three independent experiments, each evaluating 50 seeds. **(B,C)** Seedling development of *CaDIL1*-OX mutants and wild-type plants exposed to ABA. Representative images were taken 5 days after plating **(B)** and the number of seedlings in each line with expanded cotyledons was recorded **(C)**. Data represent the mean ± standard error of four independent experiments, each evaluating 25 seeds. **(D,E)** Primary root elongation of wild-type and transgenic lines exposed to ABA. Representative images were taken **(D)** and root length of each plant was measured 8 days after sowing **(E)**. Data represent the mean ± standard error of three independent experiments, each evaluating 25 seeds. **(F,G)** Primary root elongation of wild-type and transgenic plants exposed to ABA after germination. Five-day-old seedlings grown on 0.5 × MS medium were transferred to fresh 0.5 × MS medium containing 0 μM or 20 μM ABA. After 7 days, the representative images were taken **(F)**, and the root length in each line was measured **(G)**. Data represent the mean ± standard error of three independent experiments, each evaluating 25 plants. Asterisks indicate significant differences between wild-type and transgenic lines (Student’s *t*-test; *P* < 0.05).

**FIGURE 5:**
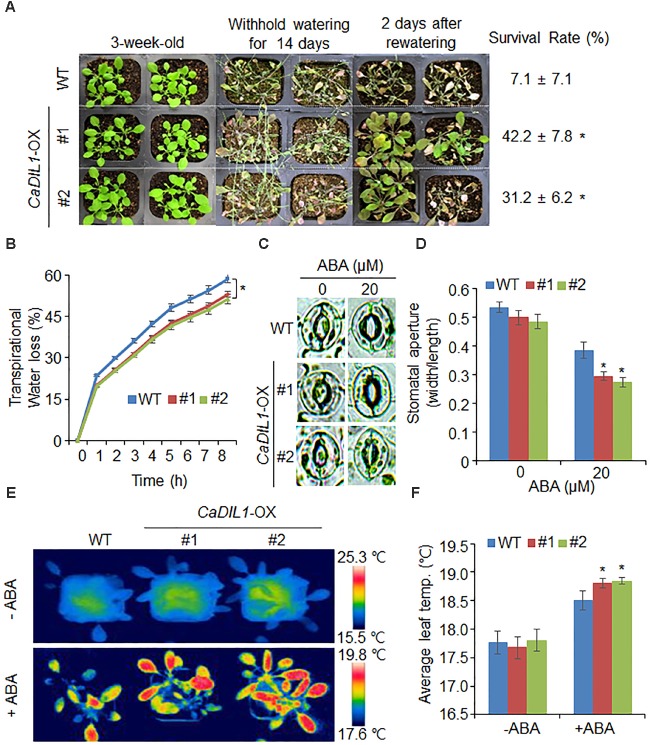
Enhanced tolerance of *CaDIL1*-OX transgenic *Arabidopsis* lines to drought stress. **(A)** Drought tolerance of *CaDIL1*-OX transgenic plants. Three-week-old wild-type (WT) and transgenic plants were subjected to drought stress by withholding watering for 14 days and rewatering for 2 days. Survival rates of plants after rewatering. Data represent the mean ± standard error of three independent experiments, each evaluating 30 plants. **(B)** Transpirational water loss from the leaves of wild-type and transgenic plants at various time points after leaf detachment. Data represent the mean ± standard error of three independent experiments, each evaluating 50 leaves. **(C,D)** Stomatal apertures in wild-type and *CaDIL1*-OX plants treated with abscisic acid (ABA). Leaf peels were harvested from 3-week-old plants of each line and incubated in stomatal opening solution containing 0 μM or 20 μM ABA. Representative images were taken under a microscope **(C)** and stomatal apertures were measured **(D)**. Data represent the mean ± standard error of three independent experiments, each evaluating 20 plants. **(E,F)** Leaf temperatures of wild-type and *CaDIL1*-OX plants exposed to ABA treatment. Leaf temperatures of each plant were measured **(E)** and representative images were taken **(F)**. Data represent the mean ± standard error of three independent experiments, each evaluating 10 plants. Asterisks indicate significant differences between wild-type and transgenic lines (Student’s *t*-test; *P* < 0.05).

*CaDIL1*-silenced pepper plants exhibited a drought-sensitive phenotype (**Figure 3**); hence, we conducted further analyses to elucidate the biological function of CaDIL1 in ABA signaling and drought stress response by using *Arabidopsis* *thaliana* that overexpressed the *CaDIL1* in the Col-0 ecotype background. The expression levels of *CaDIL1* were relatively high in T_3_ lines (*CaDIL1*-OX #1 and *CaDIL1*-OX #2) (**Supplementary Figure S1**) by RT-PCR analysis; therefore, we used these independent lines in our biological assays. Under normal growth conditions, the phenotype of *CaDIL1*-OX plants was identical to that of wild-type plants in terms of seed germination, root growth, and seedling development (**Figures 4**,**5**). To investigate the involvement of CaDIL1 in ABA signaling, we measured germination rates, cotyledon greening and primary root growth in the presence of ABA. The *CaDIL1*-OX seeds were less germinated than wild-type seeds on 0.75 μM ABA (**Figure 4A**). To evaluate ABA sensitivity in seedling stage, we determined the rates of cotyledon greening and the primary root lengths (**Figures 4B–E**). Consistent with the observed differences in germination rate, ABA treatment decreased the rates of cotyledon greening and reduced the primary root lengths. We further examined whether the altered ABA sensitivity of *CaDIL1*-OX plants at the seedling stage was an indirect response to ABA effect on seed germination or resulted directly from the ABA effect on seedling growth. To this end, 3-day-old seedlings that were germinated on Murashige and Skoog (MS) medium were transferred to MS medium containing 0 μM or 20 μM ABA (**Figures 4F,G**). As shown in **Figure 4F**, the roots of *CaDIL1*-OX seedlings were significantly shorter than those of wild-type seedlings. These data indicate that ectopic expression of *CaDIL1* confers ABA hypersensitivity in *Arabidopsis.*

### Enhanced Tolerance of *CaDIL1*-OX Plants to Drought Stress

*CaDIL1*-OX plants showed hypersensitive phenotypes to ABA; hence we investigated the drought tolerance of these plants (**Figure 5**). Under well-watered conditions, we did not observed any differences between both plants (**Figure 5A**, left panel). However, when the plants were treated with drought by withholding water for 14 days and rewatering them for 2 days (**Figure 5A**, middle and right panels, respectively), *CaDIL1*-OX plants showed a less wilted phenotypes than wild-type plants and their survival rates were higher than those of wild-type plants. To evaluate whether the drought-tolerance exhibited by *CaDIL1*-OX plants was due to increased capacity of water retention, we measured the fresh weight of rosette leaves (**Figure 5C**). The fresh weight of *CaDIL1*-OX leaves was higher than that of wild-type leaves, indicating that increased water retention contributes the drought-tolerance to *CaDIL1*-OX plants.

Different drought sensitivity is determined by several parameters, especially ABA sensitivity. Previously, our studies showed that altered stomatal aperture and leaf temperature lead to different drought sensitivity (Joo et al., 2016; Lim et al., 2017). Therefore, we determined stomatal pore size and leaf temperature in the absence and presence of ABA (**Figures 5C–F**). In the absence of ABA, we did not observed any differences in stomatal aperture or leaf temperature between both plants. However, in the presence of ABA, *CaDIL1*-OX plants showed low stomatal aperture and high leaf temperature relative to those of wild-type plants (**Figure 5C–F**). These results indicate that ectopic expression of *CaDIL1*-OX plants exhibit altered responses to drought stress.

**FIGURE 6:**
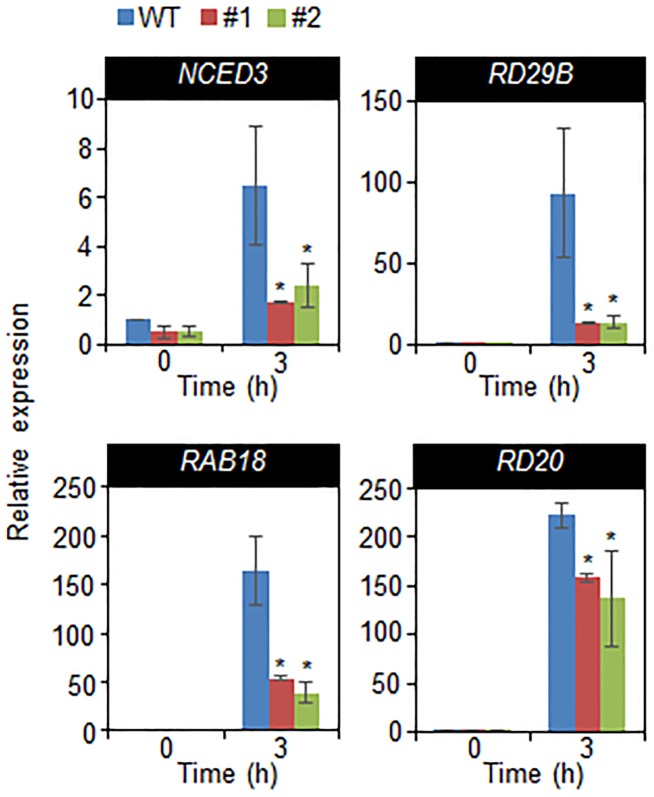
Quantitative reverse transcription polymerase chain reaction analysis of drought-inducible genes in *CaDIL1*-OX plants exposed to drought stress at 3 h after detachment. The relative expression levels (ΔΔCT) of each gene were normalized to the geometric mean of *Actin8* as an internal control gene. Data represent the mean ± standard error of three independent experiments. Asterisk indicate significant differences between wild-type and transgenic lines (Student’s *t*-test; *P* < 0.05).

Given that drought tolerance and sensitivity are correlated with expression level of stress-related genes (Gonzalez-Guzman et al., 2012; Park et al., 2015), we used quantitative RT-PCR assay to examine the expression of stress-related genes. We confirmed that the enhanced expression of *CaDIL1* altered the expression of stress-related genes in wild-type and *CaDIL1*-OX plants (**Figure 6**). When drought conditions, ABA levels are increased in various tissues, leading to induction of stress-related genes including *NCED3*, *RD29B*, *RAB18*, and *RD20.* Under normal conditions, we did not detected any different expression levels of stress-related genes between both plants. However, after 3 h of drought stress treatment, the transcripts of stress-related genes were significantly lower in *CaDIL1*-OX plants than in wild-type plants, implying that the expression of stress-related genes were affected by *CaDIL1* expression. Collectively, these results CaDIL1 protein positively modulates drought stress response via ABA-mediated signaling in plants.

## Discussion

Stomatal opening plays a crucial role in CO_2_ uptake required for photosynthesis, while stomatal closing is essential in protecting plants from drought stress. In particular, the ABA signaling pathway, which is involved in stomatal closing, can reduce transpirational water loss, thereby improving drought tolerance. Many transcription factors and enzyme-encoding genes engaged in stomatal closure have been characterized in plants, and there are many reports regarding the ABA signal transduction pathway (Hong et al., 2017; Yu et al., 2017; Huang et al., 2018; Wang et al., 2018). However, the precise mechanisms and proteins involved in stomatal opening and closing were not fully understood. Here, we isolated CaDIL1 and elucidated its function in drought stress response via ABA-mediated signaling. Under drought conditions, altered expression of *CaDIL1* in pepper and *Arabidopsis thaliana* resulted in different phenotypes by regulating ABA-induced stomatal closure.

To investigate the biological role of *CaDIL1*, we examined VIGS assay in pepper and overexpression assay in *Arabidopsis* for loss- and gain-of-function mutations, respectively. The *CaDIL1*-silenced plants exhibited reduced drought tolerance accompanied by increased transpirational water loss, indicating that a reduction in the expression of *CaDIL1* leads to a loss of the ability for stomatal closure. In contrast, *CaDIL1*-OX plants showed enhanced ABA sensitivity and drought tolerance, implying that *CaDIL1* modulates drought stress tolerance via ABA-mediated signaling. When plants encounter drought stress, endogenous ABA is rapidly synthesized and subsequently triggers stomatal closure in guard cells as the early event for preventing transpirational water loss (Schroeder et al., 2001). Several studies have suggested that enhanced stomatal closure is associated with drought tolerance (Saez et al., 2006; Aubert et al., 2010; Joo et al., 2016; Lim and Lee, 2016; Magwanga et al., 2018). Thus, we predicted a consistent pattern in ABA-treated leaves from *CaDIL1*-silenced pepper and *CaDIL1*-OX *Arabidopsis* plants. When *CaDIL1*-silenced pepper leaves were subjected to ABA treatment, the leaf temperatures were lower than those in control plants (**Figure 3F**). This pattern was opposite to that observed after ABA treatment of leaves from *CaDIL1*-OX plants (**Figure 5E**). Our data demonstrate that CaDIL1 regulates drought tolerance via ABA-induced stomatal closure.

The transcripts of stress-related genes, which are related to ABA biosynthesis and signaling pathway, are essential for adaptive response to drought stress (Zhang et al., 2006; Aubert et al., 2010; Hubbard et al., 2010; Fujita et al., 2011; Lim et al., 2015b). Based on the drought tolerance phenotypes of *CaDIL1*-OX plants, we predicted that stress-related genes are more induced in *CaDIL1*-OX plants than in wild-type plants. However, contrary to our expectations, the induction of stress-related genes were downregulated in *CaDIL1*-OX plants. If *CaDIL1*-OX plants have the processes to initiate a successful adaptive response, plants can attenuate stress and therefore are able to adapt to stress conditions; hence, the end signals of drought stress are perceived by various tissues, which then initiate a response leading to the inhibition of the expression of stress-related genes. A previous study indicated that expression of *NCED3* enhances the expression of stress-related genes; therefore, up- or downregulation of this gene influences the transcription of stress-related genes (Urano et al., 2009). Moreover, these data imply that CaDIL1 functions upstream of these stress-related genes in the drought stress response. Nevertheless, the expression level of these genes is not able to entirely explain the drought tolerant phenotypes exhibited by *CaDIL1*-OX plants.

## Conclusion

In conclusion, CaDIL1 positively regulates the ABA signaling and drought stress. *CaDIL1*-OX plants showed an ABA sensitive phenotypes in germination, seedling, and adult plant stages. The observed genetic and molecular findings provide an important insight into the plant defense response to drought stress. Nevertheless, the exact biological function whereby CaDIL1 serves as a positive component of drought stress responses remains unclear. Further studies will be necessary to find upstream and downstream of the CaDIL1, leading to elucidation of the biological role of CaDIL1 in the ABA signaling and drought stress response.

## Author Contributions

JL and CL performed experiments and analyzed the results. SL designed the experiments and wrote the manuscript.

## Conflict of Interest Statement

The authors declare that the research was conducted in the absence of any commercial or financial relationships that could be construed as a potential conflict of interest.

## Supplementary Material

FIGURE S1 **(A)** Reverse transcription-polymerase chain reaction analysis of *CaDIL1* expression in the leaves of pepper plants transfected with the empty vector control (TRV2:00) or *CaDIL1*-silenced constructs (TRV2:*CaDIL1*) 0 h after detachment. *Actin1* was used as an internal control. **(B)** Reverse transcription-polymerase chain reaction analysis of *CaDIL1* expression in wild-type (WT) and *CaDIL1*-OX transgenic lines. *Actin8* was used as an internal control gene.

TABLE S1 Sequences of primers used in this study.
