# Supplementary figures and images for: The Pepper Late Embryogenesis Abundant Protein, CaDIL1, Positively Regulates Drought Tolerance and ABA Signaling
Source: Front Plant Sci. 2018 Sep 4;9:1301. doi: 10.3389/fpls.2018.01301 (PMC6131619; doi:10.3389/fpls.2018.01301)

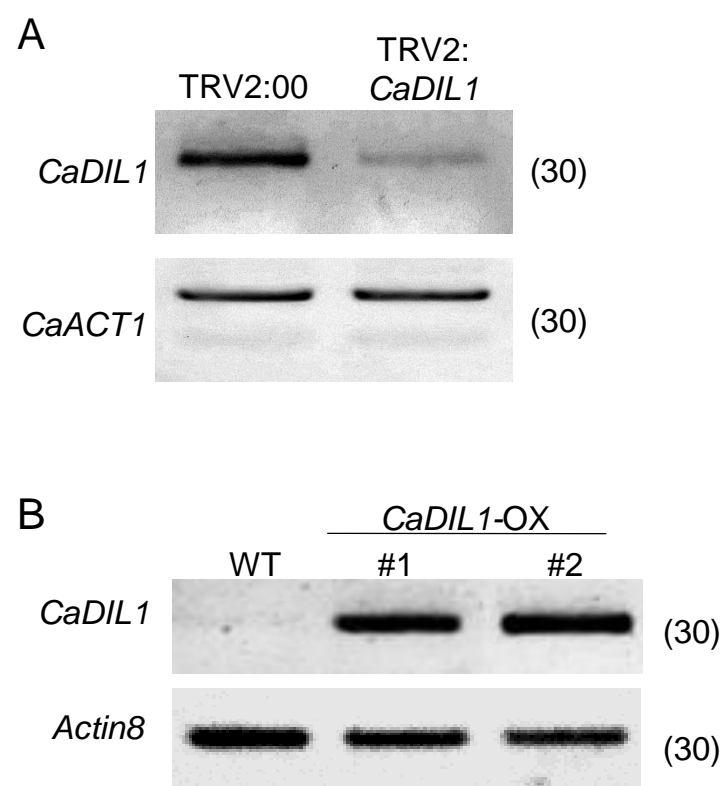

Supplement: FIGURE S1 — (A) Reverse transcription-polymerase chain reaction analysis of CaDIL1 expression in the leaves of pepper plants transfected with the empty vector control (TRV2:00) or CaDIL1-silenced constructs (TRV2:CaDIL1) 0 h after detachment. Actin1 was used as an internal control. (B) Reverse transcription-polymerase chain reaction analysis of CaDIL1 expression in wild-type (WT) and CaDIL1-OX transgenic lines. Actin8 was used as an internal control gene. [file Data_Sheet_1.PDF]
