# Supplementary material for: The Pepper Late Embryogenesis Abundant Protein, CaDIL1, Positively Regulates Drought Tolerance and ABA Signaling
Source: Front Plant Sci. 2018 Sep 4;9:1301. doi: 10.3389/fpls.2018.01301 (PMC6131619; doi:10.3389/fpls.2018.01301)
Supplement: TABLE S1 — Sequences of primers used in this study. [file Data_Sheet_2.PDF]

Supplementary Table S1. Sequences of primers used in this study

Primer name	Primer sequence (5'-3')
For cloning	
CaDIL1	Forward: ATGGCTTCCCACGAGCAGAGT<br>Reverse: CTATGGTTCTCTGGGTGTTTTTGTG
For RT-PCR	
CaDIL1	Forward: TCCACTATTCTGCTTCTCACTGAT<br>Reverse: GAAGTCCTGTCCTTTGCTTCTTGA
CaACT1	Forward: GACGTGACCTAACTGATAACCTGAT<br>Reverse: CTCTCAGCACCAATGGTAATAACTT
Actin8	Forward: CAACTATGTTCTCAGGTATTGCAGA<br>Reverse: GTCATGGAAACGATGTCTCTTTAGT
RD20	Forward: TGGTTTCCTATCTAAAGAAGCTGTG<br>Reverse: ATACAAATCCCCAAACTGAATAACA
RD29B	Forward: GTTGAAGAGTCTCCACAATCACTTG<br>Reverse: ATACAAATCCCCAAACTGAATAACA
NCED3	Forward: ACATGGAAATCGGAGTTACAGATAG<br>Reverse: AGAAACAACAAACAAGAAACAGAGC
RAB18	Forward: GGAAGAAGGGAATAACACAAAAGAT<br>Reverse: GCGTTACAAACCCTCATTATTTTTA
